# Identification of endophytic fungi with ACC deaminase-producing isolated from halophyte *Kosteletzkya Virginica*

**DOI:** 10.1080/15592324.2022.2152224

**Published:** 2022-12-04

**Authors:** Xiaomin Wang, Zengyuan Tian, Yu Xi, Yuqi Guo

**Affiliations:** a School of Life Sciences, Zhengzhou University, Zhengzhou, China; b School of Agricultural Sciences, Zhengzhou University, Zhengzhou, China

**Keywords:** Identification, endophytic fungi, ACC deaminase, *kosteletzkya virginica*

## Abstract

Seashore mallow (*Kosteletzkya virginica*), as a noninvasive perennial halophytic oilseed-producing dicot, is native from the Gulf to the Atlantic coasts of the U.S. The purpose of our research was to investigate 1-aminocyclopropane-1carboxylic acid deaminase (ACCD) producing endophytic fungi from *K.virginica*. A total of 59 endophytic fungal strains, isolated from roots in *K.virginica* of seedlings, were grouped into 12 genera including in *Penicillium, Aspergillus, Fusarium, Trichoderma, Rhizopycnis* sp., *Ceriporia Donk, Trametes* sp., *Schizophyllum commune* sp., *Alternaria, Cladosporium, Cylindrocarpon*, and *Scytalidium* according to sequences of ITS. The ACD activity of 10 endophytic fungi isolated was detected. *T.asperellum* had the highest ACC deaminase activity among all 10 isolated **genera of** fungal strains, followed by *T. viride*. **Dry weight and fresh weight of plant**, plant height, root length, SOD activity, and chlorophyll content of wheat and soybean inoculated with *T.asperellum* **or** *T. viride* was increased compared with non-inoculated control plants under non salt or salt stress. Further analysis showed that *T.asperellum* or *T.viride* strains induced downregulation of the expression of ethylene synthesis-related genes such as ACC oxidase (ACO) and ACC synthase (ACS), thereby reducing ethylene synthesis and damage to plants under salt stress. These endophytic fungi can be used as alternative bioinoculants to increase crop yield in saline soil.

## 1. Introduction

Soil salinity is frequently a limiting factor for cultivation of agricultural crops in arid and semiarid regions, which causes osmotic, ionic, and oxidative stress in plants and thus affects important morphological, physiological, and metabolic biological processes of plants, leading to serious loss of production every year.^1^ According to the FAO, 20% of the world’s irrigated and 2% of dry lands have been affected by salinity.^2^

Halophyte plants have primary and secondary mechanisms to resist salt stress. The primary mechanism focuses on increasing the intracellular osmotic pressure to expel Na^+^ from plant cells.^3^ A secondary mechanism could be the endophytic association between plant and rhizobacteria named as plant growth-promoting rhizobacteria (PGPR), which are able to improve the plant growth in abiotic stress conditions.^3^ PGPR is divided into three functional groups: plant growth-promoting bacteria (PGPB), biocontrol-PGPB, and plant stress homeo-regulating bacteria (PSHB).^4^ In the symbiotic process, some endophytic bacteria promote growth of the plants by producing siderophores, solubilizing phosphorus, secreting auxin,^5^ and synthesis of ACC deaminase (ACCD).

It was reported that when PGPR with ACCD was inoculated on the surface of rape seeds, they could absorb and utilize ACC exuded from seed coat during rape seed germination, and could effectively reduce ACC content in rape seeds and ethylene release, thus promoting root elongation and improving the salt tolerance of the seedling.^6,7^ Likewise, some endophytic fungi were used to promote plant growth and resist the stress of drought and insect pests. Through symbiotic culture of *Poplar* with a variety of fungi, it was found that these symbiotic fungi could regulate its growth and development by promoting absorption of its nutrients, and have a positive effect on the prevention of plant diseases and insect pests, help resisting salt stress.^8^ Arbuscular mycorrhizal fungi (AMF) promoted the growth and yield increase of strawberry,^9^ and thus played an important role in the growth and development of the plant.^9,10^ During this process, the endophytic fungi established a mutually beneficial relationship with the plants. Synthesizing ACCD **was** one of the important reasons for promoting plants growth.^11–13^

During exposure to salt stress, plants resulted in stress hormone ethylene. ACCD is able to catalyze the conversion of ACC, the immediate precursor of ethylene synthesis in plants, to ammonia and α-ketobutyrate and thus promotes plant growth.^7,14^ Previous studies showed that some endophytic bacteria and fungi producing ACCD not only reduced the level of ACC in plants, thereby reducing the ethylene level and mitigating biotic and abiotic stresses in plants, but also produced other biologically active substances to support the growth of their host.^15–18^ Studies have shown that PGPR with ACCD activity could decompose ACC secreted by plant roots into α-butanone acid and ammonia, which could be used as carbon source and N source for self-utilization, respectively, thereby reducing ACC concentration in root system and generating ACC concentration difference between inside and outside of root cells, thus promoting ACC secretion from root system far from short to outside and thus reducing endogenous ethylene level in plants. Inoculation with this type of PGPR could significantly reduce the negative effects of salt stress and drought stress on the growth and yield of wheat, pea, rape, tomato, and others [^19–22 23, 24^] and help plants resist the damage of tissue hypoxia.^25,26^ Therefore, PGPR with ACCD was used to minimize the impact of soil salinity on agriculture production.^27,28^

Some halophytes establish and maintain effective associations with plant growth-promoting endophytic bacteria or fungi to help them grow well in saline soil.^29^ Ethylene response factor (ERFS) is involved in abiotic and biotic stress processes, especially playing an important role under salt stress.^30^ Similarly, ***EIL***s have conservated binding sequences of plant-specific transcription factors that participate in ethylene response.^31^ In this study, endophytic fungi were isolated from seashore mallow (*K.virginica*) native in Atlantic coasts of the U.S. and their activities of ACCD were characterized. Then, the effects on wheat (*Triticum aestivum)* and soybean of these endophytic fungi were assessed. Expressions of key genes of plants, ACC synthase (ACS) and ACC oxidase (ACO), which plays an important role as the rate-limiting enzyme in ethylene synthesis induced by endophytic fungi containing the highest activities of ACCD were analyzed. And the expression levels of ***ERF***s and ***EIL***s which play an indispensable role in the corresponding pathways of ethylene signaling were **detected**. Our results showed that the promotional effect of these fungi on plant growth is attributed to their regulation on pathways of ethylene synthesis, so these endophytic fungi could be used as potential bio-inoculants to sustain crop yield production under environmental stress conditions.

## 2. Materials and methods

### 2.1. Isolation of endophytic fungi from roots of *K. virginica* seedlings

*K.virginica* seedlings were collected from **plants which were grown in** farmland alongside the Huanghe River in Zhengzhou City, Henan Province **in China**. To disinfect roots, they were rinsed with running water to get rid of the soil and dried in the air. Next, the roots of the samples were washed with distilled water and disinfected with 70% ethanol. Then, the roots were disinfected with 3% sodium hypochlorite. The **root** sections were cut into 5 mm long sections for experiments. Surface sterilization was verified by the last sterile distilled water after washing the samples, 100 µl of the final rinse water was plated on Potato Dextrose Agar (PDA) and incubated at 28°C. After thorough disinfection, hyphae or spores were removed with a dissection needle and inoculated on PDA with chloramphenicol, **purified repeatedly**, and the finally purified strain is inoculated on a slant culture medium for standby after being cultured at room temperature (25–30°C) for five days. The purified strain was inoculated onto a slope of inclined PDA medium for storage. The fungal colonies were selected according to the morphological and pigment production characteristics after incubation.^32^

### 2.2. Morphological identification of endophytic fungi

The strains were inoculated on PDA and the growth and morphological characteristics were observed. Microscopic identification: Drop a drop of sterile water in the center of the slide, pick a small **piece** of hyphae into the water with the inoculation needle, add a cover glass, and observe **with** microscope.^33^ Fourteen **species of fungal** strains colony screened were further purified on PDA medium plates and isolated single colony was transferred on **slopes of** PDA medium. **All** the isolates were preserved in **a liquid medium with** 50% glycerol at −80°C.

### 2.3. Identification of endophytic fungi

After the single colony of fungi is preliminary identified according to the standard microscopic morphology such as **pattern of** spore production, **morphology of spores, color of** aerial mycelium, texture and **shape of strain colony, their** exudates and growth rate. Isolates of fungi were incubated in PDA for 5 days at 28°C. The **genomic** DNA of isolates was extracted using CTAB method, Primer pairs ITS1(5’-TCCGTAGGTGAACCTGCGG-3’) and ITS4 (3’- TCCTCCGC TTATTGATATGC-5’) were used to amplify ribosomal internal transcribed spacer (ITS) of fungi.^34^ The thermocycler program for clone of ITS sequence is as follows: 94°C for 2 min; 40 cycles of 94°C for 30s, 55°C for 30s, and 72°C for 1 min, followed by a final extension of 72°C for 5 min. Agarose gel DNA purification kit (GE Healthcare, Buckinghamshire, UK) was used for purification of PCR products. Amplified DNA products were sequenced by Sangong (Shanghai, China). The DNA-ITS region sequence data were deposited separately to GenBank (http://blast.ncbi.nlm.nih.gov/blast.cgi) to blast their highly similar sequences.

### 2.4. Phylogenetic analyses

Multiple alignment searches were performed using the program CLUSTAL W. Phylogenetic analyses were performed by Neighbor-joining (NJ) method using MEGA 6.0.^35^

### 2.5. ACC deaminase activity

Fungi with ACCD were screened **using** Penrose,s method.^6^ Dissolved the α-ketobutyrate (Aladdin Industrial Corporation) into 0.1 M Tris-HCl pH 8.5 and made the stock solution. The solution is diluted with the same buffer to make a 0.5, 1.0, 1.5, 2.0, 2.5, 3 mmol·L^−15^ solution from which a standard concentration curve is generated. Separately, 200 μL lysate was mixed with 1.4 mL 0.56 M HCl, and 300 μL of 2,4-dinitrophenylhydrazine (Aladdin Industrial Corporation) reaction solution was added for the reaction at 30°C for 30 min, during which time the α-ketobutyrate is derivatized as a phenylhydrazone. The color of the phenylhydrazone is developed by the addition of 2.0 ml 2 M NaOH. The absorbance of the mixture was measured at 540 nm. One tube **which was not added** ACC **was** recorded as OD _(fungi extract)_, the tube of adding ACC was recorded as OD _(fungi extract+_α_−ketobutyrate)_ . OD(_α-ketobutyrate)_ will be calculated by the formula:

OD (α_−ketobutyrate)_ = OD _(fungi extract+_α_−ketobutyrate) –_ OD _(fungi extract)_ .

### 2.6. Measuring of physiological parameters of plants

*T.asperellum* and *T.viride* were grown on PDA liquid medium for 5 days. Centrifuge their fermentation to collect the hyphae and resuspend it in PBS to make hyphae suspension. The seeds of wheat (Yuguan 35) and soybean (Williams) were treated with 70% ethanol for 1 min and then with 5% sodium hypochlorite for 10 min. Then, the seeds were washed thoroughly in sterile water and cultivated with a temperature of 28/22°C and a photoperiod of 16 h/8 h (day/night) for germinating. The soybean and wheat seedlings which grow for 15 days were chosen and treated with resuspension of *T. asperellum* and *T.viride* for 5 days (5 ml/plant). **Next**, the soybean and wheat seedlings inoculated with *T.asperellum* or *T.viride* were cultivated for 15 days under the treatment of 0.5% NaCl. The experiment was conducted in the completely randomized design (CRD) with three replications. Samples were collected for analysis of phenotypic traits and activities of antioxidant enzymes.^36^

The plant height, root length, dry weight, fresh weight, and chlorophyll content were measured according to Wen’s method.^37^ The plant leaves were washed and cut into pieces, and the leaves (0.1 g) were placed in each test tubes. Acetone and anhydrous ethanol (1:1) was added and treated dark overnight until the leaves were completely white. The absorbance was measured at 663 nm and 645 nm. Chlorophyll content will be calculated by the formula:

Ca = V(12.7× OD_663_-2.69× OD_645_)/1000 w

Cb = V(22.9× OD_645_-4.68× OD_663_)/1000 w

C = Ca+Cb

V: extraction volume; w: blade weight

The SOD activity was detected with the Nitroblue tetrazolium photochemical reduction method, the fresh plant samples (0.5 g) were immediately smashed with liquid nitrogen. The samples were incubated in reaction buffer containing 5 mL of 50 mM phosphate buffer (pH 7.0), then were centrifuged for 20 min at 12000 g (4°C) . Next, the supernatant was gathered as a crude enzyme extract. The SOD activity was detected at 560 nm within 1 min.

### 2.7. Reverse Transcription Quantitative PCR (RT-qPCR) analysis of transcript abundance

RT-qPCR was performed to **analyze** the effects of *T.asperellum* and *T.viride* on *Arabidopsis Thaliana* under salt stress. First-strand cDNA synthesis was performed according to manufacturer’s instructions (HiFi-MMLV cDNA kit CW0744s). Reverse transcriptase quantitative RCR was **carried out using an Ultra SYBR Mixture ((LOW ROX) CW2601M) with Rotor-Gene (RG-3000)**. Actin was regarded as an internal control gene. The primer sequence is shown in Table 1.

**Table 1.** Primer sequences of genes used in q RT-PCR.

Gene name	Forward primer sequence (5’-3’)	Revers primer sequence (5’-3’)
At-actin	TCCCAGTGTTGTTGGTAGGC	ATGGCGACATACATAGCGGG
AT1G73730(EIL3)	GTGAGTGGTTCCTCTGACAATA	ATTTCCCAAACGCTAAACACTC
AT3G20770(EIN3)	CTGCAGATCACAACAACTTTGA	CATCCATCGTTCCTACTACTCC
AT4G17490(ERF6)	AATAATCGATCTCGTCACTCCC	GTGAAGGAATCGTTCGATTCAG
AT5G44210(ERF9)	CGAGAACGATTCTTACGTCAAC	AAAAACCAGCAGAAGCATAACC
AT2G27050(EIL1)	TTGAAGAAAGCTTGGAAAGTCG	TTTGATTGCCTCACAAGCTTAC
AT1G50640(ERF3)	GATTGTTAACCGGCCTACTAGT	CTAGGATATCTCTTCGTCGTGG
AT3G20310(ERF7)	TTATGGACCATCGGTTATACGG	TCTCTTAATCGCATGTAACGGT
AT4G28140(ERF54)	CATGATTCAAACTCGTCGGATC	TTCAGCTGGAAAAACAGAAAGG
AT2G20880(ERF53)	AGAGACATTCTGAAGCTGAGTC	GCTTCTTCTGCTGTATCGAATG
AT2G19590(ACO1)	TTGATTTTGCAGAGTTGGATGG	GAGATGAAGAAACTGCTTTCCC
AT1G06620(ACS2)	ATTCCTTCGATATTTCGTGCAC	GTTGATCACCTGGAAAAATCCC
AT1G12010(ACO3)	TGACCAACTTGAGGTGATAACA	GTAAAACGACGCGATAGACATC
AT1G62380(ACO2)	GTGAGCAATTATCCACCATGTC	GTTTCCTTCTTGTTGAGTCACC
AT5G43440(ACO12)	CTTTCACTTGCTACACTTGTCC	TAATGGCAGAGCATAAGCAAAC
AT1G03400(ACO6)	CTCATCATCTCAAGGACATGGA	GAGAAAGGAAATATCGGTGTGC
AT5G43450(ACO13)	TCATTTCGAAGATCAAAGACGC	TGTTCATGAAACCTTCGAACAC
AT2G25450(ACO7)	CATAACCCGCATGTTAACGTAG	TTAACCTTCGCAACTACACTCT
AT2G30840(ACO9)	ATGTTGGAATATGCGAAACGAG	CCCAATAACCATTCTGACGAAC
AT1G03400(ACO6)	CTCATCATCTCAAGGACATGGA	GAGAAAGGAAATATCGGTGTGC
AT1G77330(ACO5)	AAGACACTGTCTGAAATCGCTA	AGCTCAGCTTCTTCACCTTATT
AT1G05010(ACO4)	GCGATCAACTTGAGGTGATAAC	CGGAAAAATAACAGAGTCGCTT
AT5G51690(ACS12)	AGTTCCTGTAGCTAAACCTTCC	CATCTCTGTAGCAAGTCAAAGC
AT1G62960(ACS10)	GCGGCATTATAATCTCGAATCC	TCTTTCGAAAGGTCATACACGA
AT1G12010(ACO3)	TGACCAACTTGAGGTGATAACA	GTAAAACGACGCGATAGACATC
AT4G08040(ACS11)	CAGTTGTTTGAAGAGTAACGCA	CATCGTTTGGTCCGACATATTC
AT1G77330(ACO5)	AAGACACTGTCTGAAATCGCTA	AGCTCAGCTTCTTCACCTTATT
AT4G11280(ACS6)	CTGAATTCAGACAAGCTGTAGC	CGGGATTAGCTAAACAGAAAGC

### 2.8. Statistical analysis

The data were statistically analyzed using Graphpad Prism 8.0. The values are presented as mean ± standard deviation. *P*-values < 0.05 were considered significant and *P*-values < 0.01 were considered highly significant.

## 3. Results

### 3.1. Diversity of isolated endophytic fungi from *K.virginica*

**Figure1.**
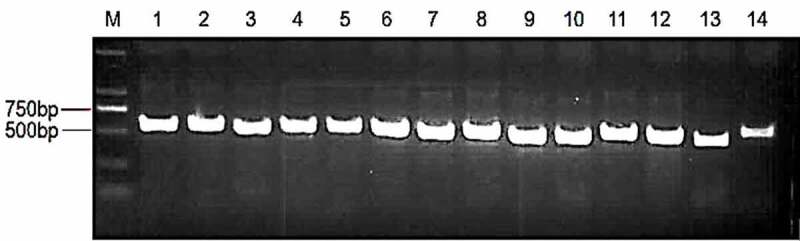
PCR Product of ITS of endophytic fungi in roots of *K. virginica* seedlings.

**Figure 2.**
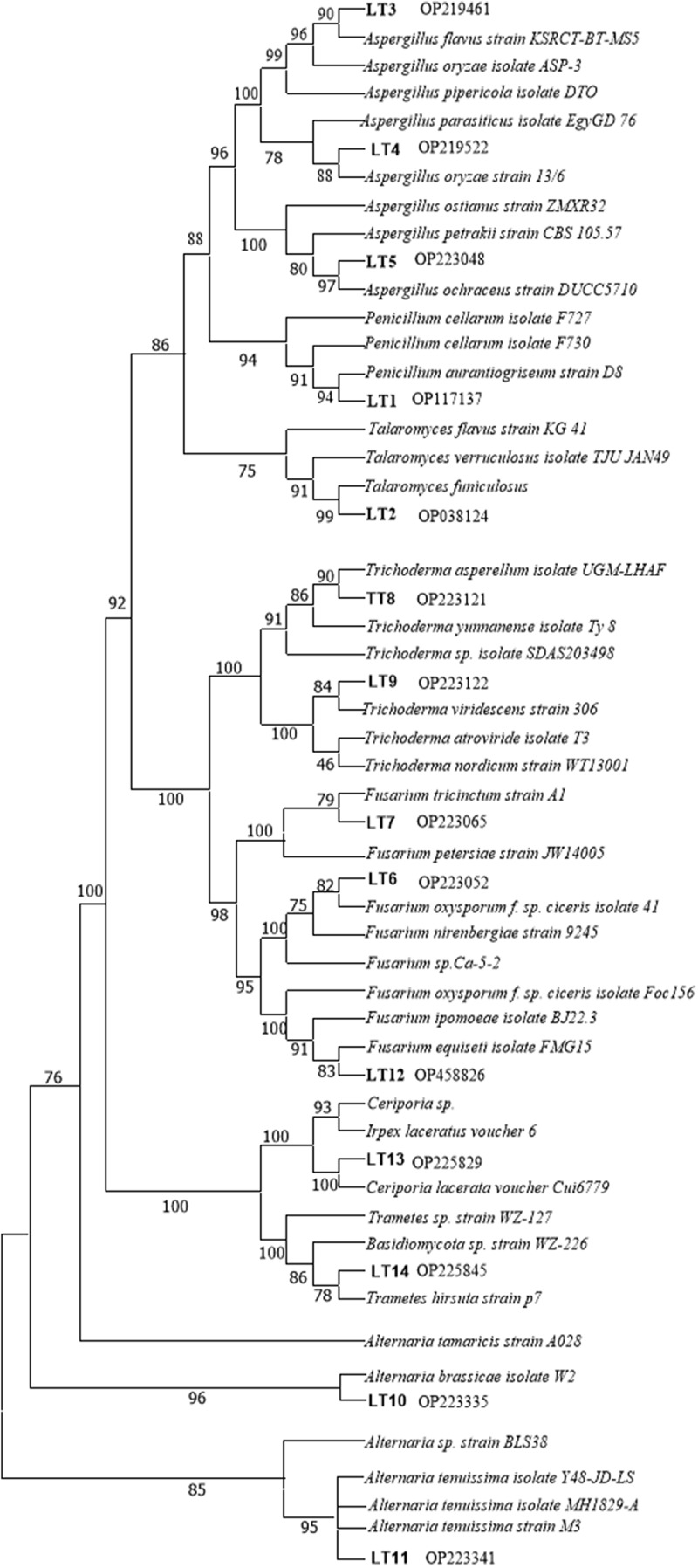
Phylogentic analysis of ITS sequences of isolated endophytic fungi (LT1~LT14) in roots of *K. virginica* seedlings. The phylogenetic analysis was performed with MEGA (6.0) using Neighbor-joining (NJ) methods with 1000 bootstrap. Values above branches represent bootstrap supports values.

Some fungi isolated from *K. virginica* were identified (Figure 1). Fourteen species of fungi (presented as LT1~ LT14) included in *penicillium aurantiogriseum, penicillium funiculosum, Aspergillus flavus, Aspergillus ochraceus, Aspergillus oryzae, Fusarium oxysporum, Fusarium tricinctum, Trichoderma asperellum, Trichoderma viride, Ceriporia lacerate, Alternaria brassicae, Alternaria tenuissima, Fusarium equiseti, Trametes hirsuta* (Table 1). The sequence data of ITS of isolated fungi was submitted to GeneBank under the accession number (OP117137~ OP458826). Molecular identification of part of the endophytic fungi from *K.virginica* was based on similarity analysis of ITS region (Figure 2).

Different genera of endophytic fungi were identified based on ITS sequences (nuclear ribosome DNA internal transcribed spacer) (shown in Table 2).

**Table 2.** Identification of endophytic fungi from roots of *K.virginica* seedlings.

Strain No	Fungal identification	Genbank accession number	Length of sequence	similarity%
LT1	*Penicillium aurantiogriseum*	OP117137	624bp	100%
LT2	*Talaromyces funiculosus*	OP038124	581bp	98.40%
LT3	*Aspergillus flavus*	OP219461	1108bp	98.87%
LT4	*Aspergillus oryzae*	OP219522	602bp	99.60%
LT5	*Aspergillus ochraceus*	OP223048	545bp	99.60%
LT6	*Fusarium oxysporum f. cubense*	OP223052	653bp	98.71%
LT7	*Fusarium tricinctum*	OP223065	562bp	100%
LT8	*Trichoderma asperellum*	OP223121	666bp	99.32%
LT9	*Trichoderma viride*	OP223122	572bp	99.65%
LT10	*Alternaria brassicae*	OP223335	591bp	99.79%
LT11	*Alternaria tenuissima*	OP223341	547bp	99.23%
LT12	*Fusarium equiseti*	OP458826	1000bp	100%
LT13	*Ceriporia lacerate*	OP225829	630bp	99.82%
LT14	*Trametes hirsuta*	OP225845	620bp	98.98%

## 3. Morphological characteristics of endophytic fungi

**Figure 3.**
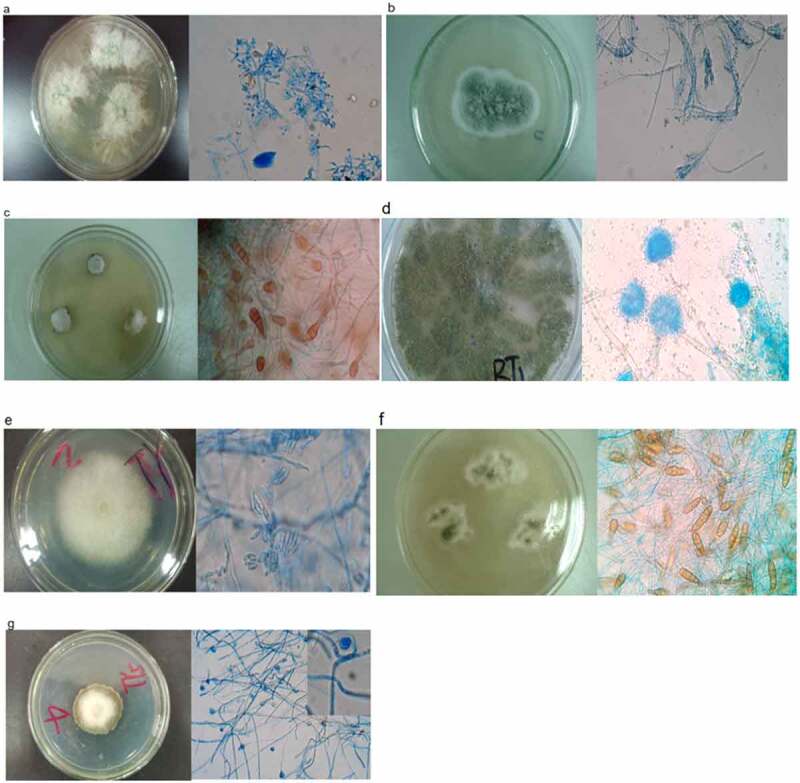
Photographs showing the gross morphology of endophytic fungi isolated from *K. virginica* grown on PDA media. a. Morphological characteristics of *T. asperellum* (LT8). b. Morphological characteristics of *T.funiculosum* (LT2). c. Morphological characteristics of *C. lacerate* (LT13). d. Morphological characteristics of *A. flavus* (LT3). e. Morphological characteristics of *F. oxysporum* (LT6). f. Morphological characteristics of *A. brassicae* (LT10). g. Morphological characteristics of *A. tenuissima* (LT11).

Due to the coevolutionary processes that occurred between plant roots and fungi, today a diverse array of fungi is able to penetrate root tissues and finally establish a symbiotic relationship with hosts. In order to understand the functions of endophytic fungi, morphology characters of their spores and mycelium of seven isolates were observed using microscope. On PDA medium plate, the diameter of the isolate of *T. asperellum* (LT8) (Figure 3a) reached 75–80 mm after five days at 25°C. At this time, it's color on the surface was white and it displayed as loose texture. After some time, the colony morphology of LT8 become flocculent, the color gradually changed into gray-green, while colorless at the bottom. Their conidia were oval, nearly colorless single cells. When gathered, they exhibited as yellow-green color, with smooth wall. The conidiospores looked like branch of the pine and cypress, which were divided into smaller alternate or opposite, bundle branches at the apex (Figure 3a). The colony of *T. funiculosum* (LT2) cultured on PDA medium displayed as velvety texture, with a diameter of 20–25 mm for five days. It then developed a dark green flocculent hyphae at the central part, accompanied with colorless exudate. The top of conidiophore gradually represented as branched structure that looked like rough broom. The shape of conidia was spherical, with nodular protrusions. The conidia chain displayed as dense and short columns (Figure 3b). The isolate (LT13) of *C. lacerate* displayed as white, thick and strong fluffy texture. The color of the colony is light yellow at the bottom of PDA medium. The hyphae were non-septum, tangled, most of which were produced on the ground (Figure 3c). The colony of *A. flavus* (LT3) grew rapidly on the medium plate. Its diameter reached 45–50 mm at 25°Cafter seven days. It displayed as loose and velvet-like texture, with indistinct radial furrows in the middle. The colony color was light green at the first stage, then grass-stained green on the surface, while slightly brown at the bottom. The conidiophore was colorless and rough. The conidia displayed a rough globular texture (Figure 3d). The colony of (LT6) *F. oxysporum* displayed as thick and flocculent texture, the hyphae were white. Color of colony was different, which was whitish, pale pink, or flesh-colored. Microconidia was oval unicellular, which clustered into pellets at the apex of conidiophore. Macroconidia took on shape of sickle. Chlamydospore produced at the apex or in the middle (Figure 3e). The colony of *A. brassicae* (LT10) grew sluggishly on the plate. The brown hyphae had substantial septate branches. The conidia spores displayed as blackish brown beaked structure, with trandiaphragm and mediastinum. There was rarely chlamydospores at the apex of dark-colored conidiophore (Figure 3f). The colony of *A. tenuissima*. (LT11) on PDA plate reached 70–75 mm in diameter at 25°C for one week. The hyphae is divided into brown, septate branches. There were few hyphaes on the ground. The conidia were long oval-shaped, which arranged in chains (Figure 3g).

### 3.1. Screening of endophytic fungi isolates for ACC deaminase activity

Plant can produce ethylene **under adverse conditions. Higher** concentration of ethylene inhibits the growth of plants. **To obtain fungi with** ACCD **which can be applied to** degrade **excessive** ethylene synthesis precursor ACC **in actual practice, we measured the** ACCD activity of **12 genera of** fungi isolated. **Their ACCD activity ranged** from about 6.82 to about 4557.92 nM α-KB mg^−15^/h. *T. asperellum* has the highest enzyme activity (4557.92 nM α-KB mg^−15^/h), followed by *T. viride* (4270.33 nM α-KB mg^−15^/h) and *A. oryzae* (1449.44 nM α-KB mg^−15^/h). The very low ACC deaminase activity was determined in strains of two genus, include *F. oxysporum*. (20.23 nM α-KB mg^−15^/h) and *P. aurantiogriseum* (6.82 nM α-KB mg^−15^/h) (Table 3).

**Table 3.** ACC deaminase activity of endophytic fungi from *K. virginica.*

Strain No	Taxonomy	ACC deaminase activity (nM α-KBmg^−15^/h)
LT1	*Penicillium aurantiogriseum*	6.82 ± 0.46
LT2	*Talaromyces funiculosus*	122.76 ± 6.11
LT3	*Aspergillus flavus*	20.36 ± 0.46
LT4	*Aspergillus oryzae*	1449.44 ± 71.54
LT5	*Aspergillus ochraceus*	482.36 ± 9.32
LT6	*Fusarium oxysporum f. cubense*	20.23 ± 0.99
LT7	*Fusarium tricinctum*	90.49 ± 34.12
LT8	*Trichoderma asperellum*	4557.92 ± 219.08
LT9	*Trichoderma viride*	4270.33 ± 119.86
LT10	*Alternaria brassicae*	264.03 ± 3.25

### 3.2. Wheat seedlings growth inoculated with *T.asperellum (Ta)* and *T.viride (Tv)* under salt stress

**Figure 4.**
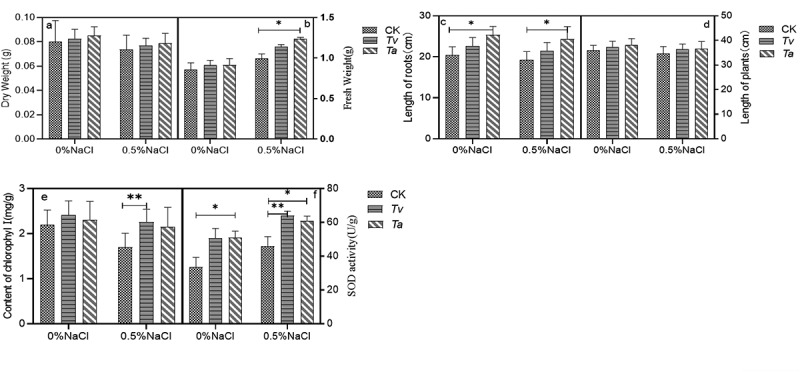
The dry weight(4a), fresh weight (4b), length of roots (4c), length of plants(4d), Content of Chlorophyll (4e) and SOD activity (4 f) in wheat seedlings inoculated with (+*Tv*/*Ta*) and without (-*Tv/Ta*). *Tv/Ta* under normal (control) and salt stress (salt).

To test the effect of endophytic fungi on the growth of plant, *T.asperellum (Ta)* and *T.viride (Tv)* with higher ACCD activity among all of isolated fungi was selected to inoculate the wheat seedlings. In normal conditions (without NaCl), the fresh weight, dry weight, root lengths, and plant height of wheat seedlings inoculated with *T.viride* increased by 5.93%, 3.32%, 11.01%, and 3.69%, respectively, compared with the control group. After inoculation with *T.asperellum*, in normal conditions (without NaCl), the fresh weight, dry weight, root lengths, and plant height, increased by 6.30%, 6.44%, 24.18%, and 5.89%, respectively. Under salt-stress conditions, the fresh weight, dry weight, root lengths, and plant height, inoculated with *T.viride* increased by significantly by 2.28%, 4.17%, 11.32%, and 4.78% compared with the control seedlings. Meanwhile, the fresh weight, dry weight, root length, and plant height inoculated with *T.asperellum* under salt-stress condition increased significantly by 17.70%, 6.85%, 26.53%, and 5.76% Figure 4(a **–** d).

The chlorophyll content in salt stress conditions was lower than that in control conditions. However, the chlorophyll content of *wheat* inoculated with *T.viride* and *T.asperellum* increased by 32.88% and 26.52% compared with that of non-inoculated under salt-stress conditions, respectively (Figure 4e). These results indicated that *T.viride* and *T.asperellum* enhanced plant tolerance to salt stress by means of chlorophyll accumulation.

Our results clearly showed that the SOD activities in the plants were higher than the activities of all other treatments (including only salt, no salt, no *T.viride* and *T.asperellum*) under salt-stress conditions (Figure 4f). Therefore, these results indicated that *T.viride* and *T.asperellum* promoted plant tolerance to salt stress through the accumulation of antioxidant enzymes.

(*Ta, Tv* represents *T.asperellum* and *T.viride* respectively). The data represents means ± standard error (SE). Different letters indicate significant differences by Turkey’s test (P value < .05).

### 3.3. Effect of Inoculation with *T.asperellum (Ta)* and *T.viride (Tv)* on soybean seedlings growth under salt stress

**Figure 5.**
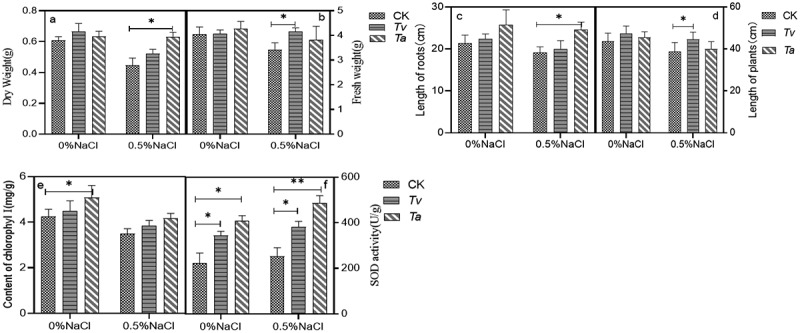
The dry weight (5a), fresh weight (5b), length of roots (5c), length of plants (5d), Content of chlorophyll (5e) and SOD activity (5 f) in wheat seedlings inoculated with (+*Tv/Ta*) and without (-*Tv/Ta*). *Tv/Ta* under normal (control) and salt stress (salt).

The root of soybean seedlings was affected by *T.asperellum* and *T.viride* under 0.5% NaCl salt stress. In normal conditions (without NaCl), the dry weight, fresh weight, root lengths and plant height of soybean seedlings inoculated with *T.viride* increased by 9.26%, 0.86%, 4.67%, and 8.52%, respectively, compared with the control group. After inoculation with *T.asperellum*, in normal conditions (without NaCl), the dry weight, fresh weight, root lengths and plant height, increased by 3.79%, 5.89%, 20.33%, and 4.35%, respectively. Under salt-stress conditions, the fresh weight, dry weight, root lengths, and plant height, inoculated with *T.viride* increased by significantly by 17.16%, 17.04%, 4.17%, and 14.80% compared with the control seedlings. Meanwhile, the fresh weight, dry weight, root length, and plant height inoculated with *T.asperellum* under salt-stress condition increased significantly by 14.56%, 26.40%, 17.97%, and 2.83% (Figure 5a-d). The results showed that *T. asperellum* and *T. viride* could significantly increase the growth of soybean seedlings under salt stress. All of them can improve the activity of SOD enzyme of soybean seedlings, enhance the oxygen-free radical scavenging system, reduce the damage of salt stress on soybean seedlings, improve the growth of soybean plants under salt stress, and enhance the tolerance of soybean to salt and the resistance of plants to abiotic stress.

(*Ta, Tv* represents *T.asperellum* and *T.viride* respectively) The data represents means ± standard error (SE). Different letters indicate significant differences by Turkey’s test (P value < .05).

### 3.4. Effect of *T. asperellum* and *T.viride* on the expression of ethylene synthesis-related genes of *A. Thaliana* under salt stress

**Figure 6.**
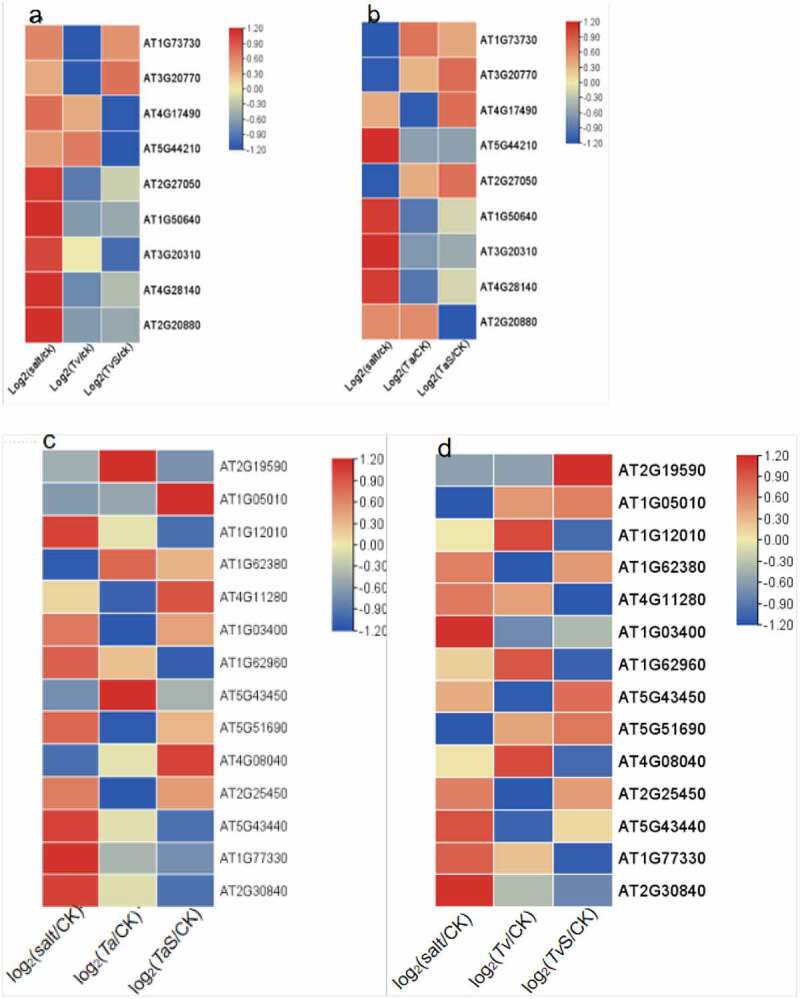
Analysis of expression pattern of ethylene synthesis-related genes (y-axis) with different treatments (x-axis). a. Expression of genes *ERF/EIL* in *A. thaliana* seedlings inoculated with *T.viride* under salt stress. b. Expression of genes *ERF/EIL* in *A. thaliana* seedlings inoculated with *T.asperellum* under salt stress. C. Expression of genes *ACO/ACS* in *A. thaliana* seedlings inoculated with *T.asperellum* under salt stress. d. Expression of genes *ACO/ACS* in *A. thaliana* seedlings inoculated with *T. viride* under salt stress.

In the ethylene biosynthesis in plants, methionine is the biosynthetic precursor of ethylene. Firstly, S-adenosyl methionine (SAM) is synthesized by converting methionine to s-adenosyl l-methionine. Then, ACC synthase (ACS), the pyridoxal enzyme, converts SAM into 1-Aminocyclopropane-1-carboxylate (ACC) and methyl-thioadenosine. ACC oxidase (ACO) finally catalyzes the conversion of ACC to ethylene, HCN, CO2, and water.^38^ A burst of reactive oxygen species (ROS) is observed within minutes after the onset of salt stress, which activates multiple signaling cascades of downstream^39^ inducing the expression of ROS-sensitive transcription factors, including ETHYLENE RESPONSE FACTORS (ERFs). As shown in (Figure 6a-6b), *AT2G27050(EIL1), AT1G50640(ERF3), AT4G28140(ERF54), AT2G20880(ERF53), AT4G17490 (ERF6),AT5G44210 (ERF9)*,and *AT3G20310 (ERF7)* in *A. Thaliana* inoculated with *T.viride*, which involved in the ethylene synthesis pathway, were down-regulated under salt stress. After inoculation with *T. aspergillus, AT3G20310(ERF7), AT1G50640(ERF3), AT4G28140(ERF54), AT5G44210 (ERF9)* and *AT2G20880(ERF53)* were significantly down-regulated under salt-stress condition. Either inoculation with *T. asperellum* or that with *T. viride*, reduced the gene expressions of *AT1G12010(ACO3), AT1G77330(ACO5)* and *AT2G30840(ACO9)*. Transcript levels of *AT2G19590(ACO1), AT5G43450(ACO13)* and *AT5G43440(ACO12)* was decreased after inoculation with *T. asperellum*. Likely, expression of *AT4G11280(ACS6), AT1G03400(ACO6), AT1G62960(ACS10), AT1G62960(ACS10)* and *AT4G08040(ACS11)* was decreased after inoculation with *T. viride*. These results indicated that the growth of plants inoculated with fungi with ACCD activity was promoted by the decrease of ethylene content under salt stress, alleviating the damage of plants.

## Discussion

Salinity stress has devastating effects on plant growth and reproduction, resulting in reduced yield.^39^ As part of the symbiosis, the plant provides certain nutrition and space of reproduction and distribution of PGPR.^40^ PGPR strains are capable of promoting plant growth by suppressing phytopathogens and alleviating abiotic stress to promote plant growth.^41^ Some PGPR with ACCD producing promoted grow of plants under salinity stress due to lowering ethylene accumulation of their hosts.^42^

In our experiment, 59 strains of fungi were isolated, and 12 genera were identified; 43 strains of bacteria were isolated from *K. virginica*, and 10 genera of bacteria were identified (data unpublished). The number of genera of fungi coexisting into *K. virginica* of roots in seedlings was more than that of bacteria. The majority of endophytic fungi were confirmed to be tolerant to salt stress. Among the fungal strains identified, *T.asperellum, T.viride, P. funiculosum, A. ochraceu*s and *F. oxysporum* were the dominant species with salt tolerance. Few of genera of fungi such as *Schizophyllum commune* and *Cladosporium sp*. were not sensitive to NaCl (data unpublished). These salt tolerant endophytic may have contributed to the growth of *K. virginica* in saline soil.

**Furthermore**, 10 fungal isolates, among 14 isolates from *K. virginica* were screened out and detected their ACCD activity. *A. flavus* generally causes significant yield losses in cereal crops due to its mycotoxin producer. However, it is also used as a beneficial fungal to promote the development of plants.^43^ *F. oxysporum* has been reported for plants growth promotion and IAA production.^44^ Previous reports have documented that *Penicillium* and *Trichoderma* possess ACCD activity,^45,46^ which was consistent with our results. ACCD hydrolyzes ACC (precursor of ethylene biosynthesis in higher plants) into alkali and α-ketobutyrate for use as a nitrogen source^28^ and enhances plant growth under saline conditions [^47^.^48^]. Likewise, ACCD can protect plants from pathogenic microorganisms and drought stress. *Trichoderma* has been shown to have promoting effect on plant growth.^49^ For example, *T. asperellum* isolated in plants grown along the marshy lands near Tarbela lake enhanced wheat tolerance under waterlogging stress.^50^ This was consistent with the fact that the, *T.asperellum* and *T.viride* obtained from *K. virginica* had ACCD in our experiment. *P. aurantiogriseum* has been reported to synthesize paclitaxel, which would facilitate metabolic engineering for the industrial production of paclitaxel from fungi.^51^ *A. ochraceus* has phosphate-solubilizing properties and disease resistance to plants. Maybe ACCD of *P. aurantiogriseum* and *A. ochraceus* derived from plants help their hosts growing in saline soils or soils in arid and semiarid regions. The photoassimilates facilitate growth, maintenance, and osmotic adjustment during physiological processes and sink organs in plants.^52^ Our research indicated inoculation with *T.asperellum* or *T. viride* increased chlorophyll of dicotyledonous soybean and monocotyledonous wheat under salt stress, implying that the two fungal isolates could counteract the suppression of photosynthesis under salt stress conditions.

Salt stress disturbs the balance of active oxygen metabolism system in plants, resulting in the production of reactive oxygen species (ROS), such as the superoxide radical(O_2_^−^), hydrogen peroxide (H_2_O_2_) and the hydroxyl radical (OH^−^), which induces membrane lipid peroxidation in plant tissues, resulting in the destruction of a series of biochemical processes and subsequently reduced plant growth.^53,54^ ROS can have adverse effect on growth and metabolism of plants. SOD was involved in scavenging ROS. Our results clearly showed that the SOD activities in the inoculated plants under salt stress conditions are higher than that of non-inoculated plants.

Earlier reports have demonstrated the role of PGPR in ameliorating salt stress through modulating the ethylene level.^12,55^ Ethylene is perceived by five receptors ETHYLENE RESPONSE1 (ETR1), ETR2, ETHYLENE INSENSITIVE4 (EIN4), ETHYLENE RESPONSE SENSOR1 (ERS1).^56,57^ ETR1 and ETR2 mediate the salt stress response through ABA and Ca2^+^pathways. In the presence of ethylene, the C-terminal of EIN2 is cut and transferred to the nucleus, where it participates in the stabilization and accumulation of EIN3/ethylene insensitive 3- like1 (EIL1), thus inducing ethylene-responsive genes.^58–60^ The ERF/EIL transcription factor, which are widely involved in the response of plants to various stresses, participated in ethylene signal pathway by binding downstream gene promoters to initiate their gene expression.^57,61^ EIL proteins are the key components of ethylene signal transduction,^31^ which play an important role in plant response to abiotic stress. A clear coexpression was observed between ERF6 and ERF11 under numerous biotic and abiotic stress conditions.^62^ In our experimental records, the expression level of *AT4G17490 (ERF6)* inoculated with *T. viride* was remarkably decreased compared with that without inoculation under salt stress. The expression levels of *AT1G50640(ERF3)*, and *AT5G44210 (ERF9)* were significantly increased under both biotic and abiotic stresses.^63^ Expression of *AT1G50640 (ERF53)* and *AT4G28140 (ERF54)* have been induced in plant responses to cold and heat stress as well as lateral root development.^64,65^ Here, they were significantly reduced after inoculation with *T. viride* and *T.asperellum* under salt stress in our experiment. ERF and EIL genes were down regulated in *Arabidopsis* seedlings inoculated with *T.asperellum* or *T. viride*. These results indicated inoculation with *T.asperellum* and *T. viride* decreased ethylene synthesis, thus improved the tolerance of seedlings to salt stress.

In the process of ethylene biosynthesis, ACO and ACS are important rate-limiting enzymes AtERF11 can interact with the ACS2/5 gene promoter to inhibit the expression of ACS2 and ACS5 genes, thereby negatively regulating the synthesis of ethylene. The regulation of ACS activity and ACC content in the cellular pathway ultimately determines the intracellular ethylene synthesis rate.^31,66^ Previous studies have also demonstrated that under high salinity, ethylene level increased due to enhanced ACO activity.^67,68^ After inoculation with *T.asperellum*, expression of ACO gene was reduced, which could be due to decrease in ACC content caused by activities of ACCD in the fungal isolates.

In conclusion, this study reported firstly on isolation and characterization of endophytic fungi of roots in *K. virginica* seedlings. Some fungal isolates with ACCD activity could be used to enhance salt tolerance of glycophytes in saline soil.

## Date availability statement

All data that support the findings reported in this study are available from the corresponding author upon reasonable request.

## References

[1] Munns R, Gilliham M. Salinity tolerance of crops–what is the cost? New Phytol. 2015;208:668–672–12. 26108441 10.1111/nph.13519

[2] Salwan R, Sharma A, Sharma V. Microbes mediated plant stress tolerance in saline agricultural ecosystem. Plant Soil. 2019;442:1–22.

[3] Blumwald E. Sodium transport and salt tolerance in plants. Current Opinion in Cell Biology. 2000;12(4):431–434. doi:10.1016/S0955-0674(00)00112-5. 10873827

[4] Bashan Y, Holguin G. Proposal for the division of plant growth-promoting rhizobacteria into two classifications: biocontrol-PGPB (plant growth-promoting bacteria) and PGPB. S.b.b. 1998;30:1225–1228.

[5] Zhao L, Wang Y, Kong S. Effects of Trichoderma asperellum and its siderophores on endogenous auxin in Arabidopsis thaliana under iron-deficiency stress. Int. Microbiol. 2020;23:501–509. 32080772 10.1007/s10123-020-00122-4

[6] Penrose DM, Glick BR. Methods for isolating and characterizing ACC deaminase-containing plant growth-promoting rhizobacteria. Physiol Plant. 2003;118:10–15. 12702008 10.1034/j.1399-3054.2003.00086.x

[7] Rashid S, Charles TC, Glick BR. Isolation and characterization of new plant growth-promoting bacterial endophytes. Appl. Soil Ecol. 2012;61:217–224.

[8] Derksen H, Rampitsch C, Daayf F. Signaling cross-talk in plant disease resistance. Plant Sci. 2013;207:79–87. doi:10.1016/j.plantsci.2013.03.004. 23602102

[9] Guerrero-Molina MF, Winik BC, Pedraza RO. More than rhizosphere colonization of strawberry plants by *Azospirillum brasilense*. Applied Soil Ecology. 2012;61:205–212. doi:10.1016/j.apsoil.2011.10.011.

[10] Ipek M, Pirlak L, Esitken A, Figen Dö Nmez M, Turan M, Sahin F. Plant growth-promoting rhizobacteria (Pgpr) increase yield, growth and nutrition of strawberry under high-calcareous soil conditions. Journal of Plant Nutrition. 2014;37(7):990–1001. doi:10.1080/01904167.2014.881857.

[11] Douriet-Gámez NR, Maldonado-Mendoza IE, Ibarra-Laclette E, Blom J, Calderó n-Vá Zquez CL. Genomic analysis of Bacillus sp. strain B25, a biocontrol agent of maize pathogen *Fusarium verticillioides*. Curr Microbiol. 2018;75(3):247–255. doi:10.1007/s00284-017-1372-1. 29051980

[12] Misra S, Dixit VK, Khan MH, Kumar MS, Dviwedi G, Yadav S, Lehri A, Singh Chauhan P. Exploitation of agro-climatic environment for selection of 1-aminocyclopropane-1-carboxylic acid (ACC) deaminase producing salt-tolerant indigenous plant growth promoting rhizobacteria. Microbiol Res. 2017;205:25–34. 28942841 10.1016/j.micres.2017.08.007

[13] Wang WF, Wu ZS, He YH, Huang YY, Li X, Ye BC. Plant growth promotion and alleviation of salinity stress in *Capsicum annuum L*. by *Bacillus* isolated from saline soil in Xinjiang. Ecotoxicol Environ Saf. 2018;164:520–529. 30149350 10.1016/j.ecoenv.2018.08.070

[14] Glick BR. Bacteria with ACC deaminase can promote plant growth and help to feed the world. Microbiol. Res. 2014;169(1):30–39. doi:10.1016/j.micres.2013.09.009. 24095256

[15] Arnold AE, Henk DA, Eells RL, Lutzoni F, Vilgalys R. Diversity and phylogenetic affinities of foliar fungal endophytes in loblolly pine inferred by culturing and environmental PCR. Mycologia. 2007;99(2):185–206. doi:10.1080/15572536.2007.11832578. 17682771

[16] Compant S, Samad A, Faist H, Sessitsch A. A review on the plant microbiome: ecology, functions, an emerging trends in microbial application. J. Adv. Res. 2019;19:29–37. doi:10.1016/j.jare.2019.03.004. 31341667 PMC6630030

[17] Glick BR. Modulation of plant ethylene levels by the bacterial enzyme ACC deaminase. FEMS Microbiol. Lett. 2005;251(1):1–7. doi:10.1016/j.femsle.2005.07.030. 16099604

[18] Saxena S. Microbial metabolites for development of ecofriendly agrochemical. Allelopathy J. 2014;33:1–23.

[19] Arshad M, Shaharoona B, Mahmood T. Inoculation with *Pseudomonas* spp. containing ACC-deaminase partially eliminates the effects of drought stress on growth, yield, and ripening of pea (*Pisum sativum* L.). Pedosphere. 2008;18(5):611–620. doi:10.1016/S1002-0160(08)60055-7.

[20] Jalilia F, Khavazib K, Pazirac E, Nejati A, Rahmani HA, Sadaghiani HR, Miransari M. Isolation and characterization of ACC deaminase-producing fluorescent pseudomonads, to alleviate salinity stress on canola (*Brassica napus* L.) growth. Journal of Plant Physiology. 2009;166(6):667–674. doi:10.1016/j.jplph.2008.08.004. 18829132

[21] Sheehy RE, Honma M, Yamada M, Sasaki T, Martineau B, Hiatt WR. Isolation, sequence, and expression in Escherichia coli of the *Pseudomonas* sp. Strain ACP gene encoding 1-aminocyclopropane-1-carboxylate deaminase. J. Bacteriol. 1991;173:5260–5265. 1885510 10.1128/jb.173.17.5260-5265.1991 PMC208234

[22] Mayak S, Tirosh T, Glick BR. Plant growth-promoting bacteria that confer resistance to water stress in tomatoes and peppers. Plant Sci. 2004;166:525–530.

[23] Ramadoss D, Lakkineni VK, Bose P, Ali S, Annapurna K. Mitigation of salt stress in wheat seedlings by halotolerant bacteria isolated from saline habitats. SpringerPlus. 2013;2:6. 23449812 10.1186/2193-1801-2-6 PMC3579424

[24] Zahir ZA, Ghani U, Naveed M, Nadeem SM, Asghar HN. Comparative effectiveness of *Pseudomonas* and *Serratia* sp. containing ACC-deaminase for improving growth and yield of wheat (*Triticum aestivum* L.) under salt-stressed conditions. Arch. Microbiol. 2009;191:415–424. 19255743 10.1007/s00203-009-0466-y

[25] Barnawal D, Bharti N, Maji D, Chanotiya CS, Kalra A. 1-Aminocyclopropane-1-carboxylic acid (ACC) deaminase-containing rhizobacteria protect Ocimum sanctum plants during waterlogging stress via reduced ethylene generation. Plant Physiology and Biochemistry. 2012;58:227–235. doi:10.1016/j.plaphy.2012.07.008. 22846334

[26] Li J, McConkey BJ, Cheng ZY, Guo SR, Glick BR. Identification of plant growth-promoting bacteria-responsive proteins in cucumber roots under hypoxic stress using a proteomic approach. Journal of Proteomics. 2013;84:119–131. doi:10.1016/j.jprot.2013.03.011. 23568019

[27] Ahmad M, Zahir A, Asghar Z, Asghar M. Inducing salt tolerance in mung bean through coinoculation with rhizobia and plant-growth-promoting rhizobacteria containing 1-aminocyclopropane 1- carboxylate deaminase. Can. J Microbiol. 2011;57(7):578–589. doi:10.1139/w11-044. 21770816

[28] Glick BR, Penrose DM, Li J. A model for the lowering of plant ethylene concentrations by plant growth-promoting bacteria. Journal of Theoretical Biology. 1998;190(1):63–68. doi:10.1006/jtbi.1997.0532. 9473391

[29] Sgroy V, Cassán F, Masciarelli O, Papa MFD, Lagares A, Luna V . Isolation and characterization of endophytic plant growth-promoting (PGPB) or stress homeostasis-regulating (PSHB) bacteria associated to the halophyte Prosopis strombulifera. Appl. Microbiol. Bio. 2009;85(2):371–381. 10.1007/s00253-009-2116-3. 19655138

[30] Han DG, Han JX, Yang GH, Wang S, Xu TL, Li WH. An ERF transcription factor gene from malus baccata (l.) borkh, mberf11, affects cold and salt stress tolerance in arabidopsis. Forests. 2020;11(5):514. doi:10.3390/f11050514.

[31] Liu CY, Li J, Zhu PP, Yu J, Hou JM, Wang CH, Long DP, Yu MD, Zhao AC. Mulberry EIL3 confers salt and drought tolerances and modulates ethylene biosynthetic gene expression. PEERJ. 2019;7:e6391. doi:10.7717/peerj.6391. 30809434 PMC6385683

[32] Sunitha VH, Nirmala Devi D, Srinivas C. Extracellular enzymatic activity of endophytic fungal strain isolated from medicinal plants. World J. Agri. Sci. 2013;9:01–09.

[33] Paul NC, Deng JX, Shin KS, Yu SH. Molecular and Morphological Characterization of EndophyticHeterobasidion araucariae from Roots ofCapsicum annuum L. in Korea. Mycobiology. 2018;40:85–90. 10.5941/MYCO.2012.40.2.85 PMC3408308 22870048

[34] Li G-R, Cao B-H, Liu W, Ren R-H, Feng J, Lv D-J. Isolation and identification of endophytic fungi in kernels of coix lachrymal-jobi l. cultivars. Current Microbiology. 2020;77(8):1448–1456. doi:10.1007/s00284-020-01950-3. 32198535

[35] Tamura K. MEGA5: molecular evolutionary genetics analysis using maximum likelihood, evolutionary distance, and maximum parsimony methods. Mol. Biol. Evol. 2011;28:2731–2739. 21546353 10.1093/molbev/msr121 PMC3203626

[36] Demiral T, Türkan I. Comparative lipid peroxidation, antioxidant defense systems and proline content in roots of two rice cultivars differing in salt tolerance. Environ. Exp. Bot. 2005;53(3):247–257. doi:10.1016/j.envexpbot.2004.03.017.

[37] Adil M, Bashir S, Aslam Z, Ahmad N, Alkahtni J, Bashir S, Asghar RMA, Dwiningsih Y, Younas T, Elshikh MS. Zinc oxide nanoparticles improved chlorophyII contents,physical parameters, and wheat yield under salt stress. Front. Plant Sci. 2022;13:932861. doi:10.3389/fpls.2022.932861 PMC9382196 35991444

[38] Orozco-Mosqueda MDC, Glick BR, Santoyo G. ACC deaminase in plant growth-promoting bacteria (PGPB): an efficient mechanism to counter salt stress in crops. Microbiol Res. 2020;235:126439. 32097862 10.1016/j.micres.2020.126439

[39] Isayenkov SV, Maathuis FJM. Plant Salinity Stress: many Unanswered Questions Remain. Front Plant Sci. 2019;10:80. doi:10.3389/fpls.2019.00080. 30828339 PMC6384275

[40] Schardl CL, Leuchtmann A, Spiering MJ. Symbioses of Grasses with Seedborne Fungal Endophytes. Annu. Rev. Plant Biol. 2004;55:315–340. 15377223 10.1146/annurev.arplant.55.031903.141735

[41] Lu P, Jiang K, Hao YQ, Chu WY, Xu YD, Yang JY, Chen JL, Zeng GH, Zhao HX. Profiles of bacillus spp. isolated from the rhizosphere of suaeda glauca and their potential to promote plant growth and suppress fungal phytopathogens. J. Microbiol and Biotechn. 2021;31(9):1231–1240. 10.4014/jmb.2105.05010 PMC9706026 34261851

[42] Naing AH, Maung TT, Kim CK. The ACC deaminase‐producing plant growth‐promoting bacteria: influences of bacterial strains and ACC deaminase activities in plant tolerance to abiotic stress. Physiol Plantarum. 2021;173:1992–2012. 10.1111/ppl.13545 34487352

[43] Lubna, Asaf S, Hamayun M, Khan AL, Waqas M, Khan MA, Jan R, Lee IJ, Hussain A. Salt tolerance of Glycine max. L induced by endophytic fungus Aspergillus flavus CSH1, via regulating its endogenous hormones and antioxidative system. Plant Physiol Bioch. 2018;128:13–23. 10.1016/j.plaphy.2018.05.007 29751251

[44] Deng ZJ, Cao LX. Fungal endophytes and their interactions with plants in phytoremediation: a review. Chemosphere. 2017;168:1100–1106. doi:10.1016/j.chemosphere.2016.10.097. 28029384

[45] Hontzeas N, Richardson AO, Belimov A, Safronova V, Abu-Omar MM, Glick BR. Evidence for horizontal transfer of 1-aminocyclopropane-1-carboxylate deaminase genes. Applied and Environmental Microbiology. 2005;71(11):7556–7558. doi:10.1128/AEM.71.11.7556-7558.2005. 16269802 PMC1287689

[46] Yim W, Seshadri S, Kim K, Lee G, Sa C. Ethylene emission and PR protein synthesis in ACC deaminase producing Methylobacterium spp. inoculated tomato plants (Lycopersicon esculentum Mill.) challenged with *Ralstonia solanacearum* under greenhouse conditions. Plant Physiol. Biochem. 2013;67:95–104. 23558008 10.1016/j.plaphy.2013.03.002

[47] Singh PR, Shelke GM, Kumar A, Jha PN. Biochemistry and genetics of ACC deaminase: a weapon to “stress ethylene” produced in plants. Front. Microbiol. 2015;6:1225. 26441873 10.3389/fmicb.2015.00937 PMC4563596

[48] Vives-Peris V, Gómez-Cadenas A, Pérez-Clemente RM. Salt stress alleviation in citrus plants by plant growth-promoting rhizobacteria *Pseudomonas putida* and *Novosphingobium* sp. Plant Cell Rep. 2018;37:1557–1569. 30062625 10.1007/s00299-018-2328-z

[49] Harman GE, Doni F, Khadka RB, Uphoff N. Endophytic strains of Trichoderma increase plants’ photosynthetic capability. Journal of Applied Microbiology. 2021;130(2):1–18. doi:10.1111/jam.14368. 31271695

[50] Rauf M, Awais M, Ud-Din A, Ali K, Gul H, Rahman MM, Hamayun M, Arif M. Molecular mechanism of the 1-Aminocyclopropane-1-Carboxylic Acid (ACC) deaminase producing Thichoderma asperellum MAP1 in enhancing wheat tolerance to waterlogging stress. Front. Plant Sci. 2021;11:614971. 33537050 10.3389/fpls.2020.614971 PMC7847992

[51] Yang YF, Zhao HN, Barrero RA, Zhang BH, Sun GL, Wilson LW, Xie FL, Walker KD, Parks JW, Bruce R, Guo GW, Chen L, Zhang Y, Huang X, Tang Q, Liu HW, Bellgared M, Qiu D, Lai JS. Genome sequencing and analysis of the paclitaxel-producing endophytic fungus Penicillium aurantiogriseum NRRL 62431. BMC Genomics. 2014;15:69. doi:10.1186/1471-2164-15-69. 24460898 PMC3925984

[52] Munns R, Termaat A. Whole-plant responses to salinity. Funct Plant Biol. 1986;13:143–160.

[53] Jan M, Shah G, Masood S, Shinwari KI, Hameed R, Rha ES, Jamil M. . *Bacillus cereus* enhanced phytoremediation ability of rice seedlings under cadmium toxicity. Biomed Res. Int. 2019:8134651. Accessed 24 July, 2019. 10.1155/2019/8134651 PMC6681586 31428647

[54] Khan MA, Asaf S, Khan AL, Adhikari A, Jan R, Ali S, Imran M, Kim K-M, Lee I-J, Papen H. Plant growth-promoting endophytic bacteria augment growth and salinity tolerance in rice plants. Plant Biology. 2020;22(5):850–862. doi:10.1111/plb.13124. 32329163

[55] Bal H, Nayak L, Das S, Adhya T. Isolation of ACC deaminase producing PGPR from rice rhizosphere and evaluating their plant growth-promoting activity under salt Stress. Plant Soil. 2013;366(1–2):93–105. doi:10.1007/s11104-012-1402-5.

[56] Depaepe T, Hendrix S, Janse van Rensburg HC, Vanden Ende W, Cuypers A, Van Der Straeten D. At the crossroads of survival and death: the reactive oxygen species-ethylene-sugar triad and the unfolded protein response. Trends Plant Sci. 2021;26(4):338–351. doi:10.1016/j.tplants.2020.12.007. 33431325

[57] Wilson RL, Kim H, Bakshi A. The ethylene receptors ETHYLENE RESPONSE1 and ETHYLENE RESPONSE2 have contrasting roles in seed germination of Arabidopsis during salt stress. Plant Physiol. 2014;165:1353–1366. 24820022 10.1104/pp.114.241695 PMC4081342

[58] Ju C, Yoon GM, Shemansky JM, Lin DY, Ying ZI, Chang J, Garrett WM, Kessenbrock M, Groth G, Tucker ML, ***et al***. CTR1 phosphorylates the central regulator EIN2 to control ethylene hormone signaling from the ER membrane to the nucleus in Arabidopsis. PNAS. 2012;109(47):19486–19491. doi:10.1073/pnas.1214848109. 23132950 PMC3511113

[59] Qiao H, Shen ZX, Huang SC, Schmitz RJ, Urich MA, Briggs SP, Ecker JR. Processing and subcellular trafficking of ER-tethered EIN2 control response to ethylene gas. Sci. 2012;338:390–393. 10.1126/science.1225974 PMC3523706 22936567

[60] Wen X, Zhang C, Zhao Q, Ji YS, He WR, An FY, Jiang LW, Guo HW. Activation of ethylene signaling is mediated by nuclear translocation of the cleaved EIN2 carboxyl terminus. Cell Res. 2012;22:1613–1616. 23070300 10.1038/cr.2012.145 PMC3494400

[61] Jiang M, Ye Z-H, Zhang H-J, Miao L-X. Broccoli plants over-expressing an ERF transcription factor gene BoERF1 facilitates both salt stress and *Sclerotinia* stem rot resistance. J Plant Growth Regul. 2019;38(1):1–13. doi:10.1007/s00344-018-9799-6.

[62] Hruz T, Laule O, Szabo G, Wessendorp FB, Oertle S, Widmayer L, Gruissem P, Zimmermann P. Genevestigator V3: a reference expression database for the meta-analysis of transcriptomes. Advance in Bioinformatics. 2008;2008:35–39. 10.1155/2008/420747 PMC2777001 19956698

[63] Zhang ZY, Yao WLD, Liang N, Liu HX, Huang RF. A novel ERF transcription activator in wheat and its induction kinetics after pathogen and hormone treatments. J. Exp Bot. 2007;58:2993–3003. 17728298 10.1093/jxb/erm151

[64] Templalexis D, Tsitsekian D, Liu C. Potassium transporter TRH1/KUP4 contributes to distinct auxin-mediated root system architecture responses. Plant Physiol. 2022;188:1043–1060. 34633458 10.1093/plphys/kiab472 PMC8825323

[65] Wang CG, Zhang MY, Zhou JJ. Transcriptome analysis and differential gene expression profling of wucai (Brassica campestris L.) in response to cold stress. BMC Genomics. 2022;23:127. 35168556 10.1186/s12864-022-08311-3 PMC8848729

[66] Sofy MR, Aboseidah AA, Heneidak SA, Ahmed HR. ACC deaminase containing endophytic bacteria ameliorate salt stress in *Pisum sativum* through reduced oxidative damage and induction of antioxidative defense systems. Environ Sci Pollut Res. 2021;28:40971–40991. 10.1007/s11356-021-13585-3 33772716

[67] Kukreja S, Nandwal AS, Kumar N, Sharma SK, Sharma SK, Unvi V, Sharma PK. Plant water status, H_2_O_2_ scavenging enzymes, ethylene evolution and membrane integrity of Cicer arietinum roots as affected by salinity. Biologia plantarum. 2005;49(2):305–308. doi:10.1007/s10535-005-5308-4.

[68] Peng Z, He SP, Gong WF, Sun JL, Pan ZE, Xu FF, Lu YL, Du XM. Comprehensive analysis of differentially expressed genes and transcriptional regulation induced by salt stress in two contrasting cotton genotypes. BMC Genomics. 2014;15:760. 25189468 10.1186/1471-2164-15-760 PMC4169805

